# Proposal of Environmental Impact Assessment Method for Concrete in South Korea: An Application in LCA (Life Cycle Assessment)

**DOI:** 10.3390/ijerph13111074

**Published:** 2016-11-02

**Authors:** Tae Hyoung Kim, Sung Ho Tae

**Affiliations:** 1 Building and Urban Research Institute, Korea Institute of Civil Engineering and Building Technology, Daehwa-dong 283, Goyandae-Ro, ILsanseo-Gu, Goyang-Si 10223, Gyeonggi-Do, Korea; kimtaehyoung@kict.re.kr; 2 School of Architecture & Architectural Engineering, Hanyang University, 1271 Sa 3-dong, Sangrok-Gu, Ansan-Si 15588, Gyeonggi-Do, Korea

**Keywords:** concrete, life cycle assessment, environmental impact, South Korea

## Abstract

This study aims to develop a system for assessing the impact of the substances discharged from concrete production process on six environmental impact categories, i.e., global warming (GWP), acidification (AP), eutrophication (EP), abiotic depletion (ADP), ozone depletion (ODP), and photochemical oxidant creation (POCP), using the life a cycle assessment (LCA) method. To achieve this, this study proposed an LCA method specifically applicable to the Korean concrete industry by adapting the ISO standards to suit the Korean situations. The proposed LCA method involves a system that performs environmental impact assessment on the basis of input information on concrete mix design, transport distance, and energy consumption in a batch plant. The Concrete Lifecycle Assessment System (CLAS) thus developed provides user-friendly support for environmental impact assessment with specialized database for concrete mix materials and energy sources. In the case analysis using the CLAS, among the substances discharged from the production of 24 MPa concrete, those contributing to GWP, AP, EP, ADP, ODP, and POCP were assessed to amount to 309 kg-CO_2_ eq/m^3^, 28.7 kg-SO_2_ eq/m^3^, 5.21 kg-PO_4_^3−^ eq/m^3^, 0.000049 kg-CFC_11_ eq/m^3^, 34 kg/m^3^, and 21 kg-Ethylene eq/m^3^, respectively. Of these six environmental impact categories selected for the LCA in this study, ordinary Portland cement (OPC) was found to contribute most intensely to GWP and POCP, and aggregates, to AP, EP, ODP, and ADP. It was also found that the mix design with increased prop proportion of recycled aggregate was found to contribute to reducing the impact in all other categories.

## 1. Introduction

The ground granulated blast furnace slag (GGBS) portion of concrete has reduced global warming potential (GWP), but it continues to have similar or even increased impact on other environmental impact categories. In contrast, while GWP increased as the recycled aggregate mix ratio increased, the mix design with increased GWP is still one of the key materials in the construction industry. It releases a large amount of environmentally hazardous substances into the atmosphere throughout its life cycle from production to construction, maintenance/management, and demolition/waste management. Technologies to assess and reduce its environmental impact should hence be developed by studying its impact under the life cycle aspect.

Energy consumption continues when cement and aggregate raw materials are transported to concrete manufacturers (batch plants), where concrete is produced, whereby various environmentally hazardous substances are released into the atmosphere, leading to air, water, and soil contamination. Due to the variety of production process and continuous environmental loading throughout its life cycle, the environmental impact of concrete should be assessed in relation to various environmental impact categories. In this regard, public institutions in environmentally advanced countries, such as the Building Research Establishment (BRE) [1] and the Swedish Environmental Management Council (SEMCO) [2], have developed Product Category Rules (PCRs) on environmental declarations for the construction materials from production to disposal and are administering relevant certification systems. Currently, research projects are underway on the concrete-specific international standards ISO13315-2 (Environmental management for concrete and concrete structures) [3]. Unfortunately, pertinent research efforts in Korea tend to be focused on GWP and assess only greenhouse gas (GHG) emissions, paying little attention to the development of PCRs and standards for other environmental impact categories such as acidification, ozone depletion, and eutrophication. Such a single-category environmental impact assessment cannot yield any comprehensive concrete-specific assessment results.

In an attempt to improve this drawback, this paper presents a life cycle assessment (LCA) method tailored to Korean concrete industry based on the existing international standards on LCA, ISO 14025 [4]/14044 [5], and ISO 21930 [6]. The functional unit and system boundary of the proposed concrete-specific LCA were defined. The scope of assessment was selected to cover the steps of raw material extraction, transportation, and concrete production. Analysis was performed on the input and output materials in each step. Furthermore, applying the proposed concrete LCA method, this study developed the Concrete Lifecycle Assessment System (CLAS), and performed a case study.

## 2. Analysis of Previous Studies

As shown in Table 1, this study analyzed the assessment elements and methods of LCA systems developed at home and abroad. South Korea’s representative LCA systems are TOTAL (Tool for Type 3 labeling And Lca) [7], PASS (Product Assessment for Sustainable Solutions) [8], COOL [9], etc. All of them perform environmental impact assessment on the basis of the National Life Cycle Index (LCI) Database [10]. The LCI Database for Korean industry contain standard data established by cataloguing the average input of natural resources during the entire production process of every product in all industrial activities, such as primary materials, transport, processing, and disposal, as well as the corresponding emissions to the environment and waste generation. Additionally, these systems were developed to enable LCAs of all industrial products, whereby the overall production process flow can be drafted, life cycle data inventory can be compiled by selecting the LCI database for each unit process, and impact assessment can be performed for six environmental impact categories. However, they were analyzed to have limitations of insufficient database for unit loading factor of raw materials necessary for a concrete LCA and inter-rater inconsistencies in input information calculation methods for the requirement of standard data collection methods and scope of assessment in different production sites. Representative LCA systems at international level include ATHENA Impact Estimator [11], Gabi Build-it [12], BEES (Building for Environmental and Economic Sustainability) [13], and Sima-Pro [14].

The analyses of these domestic and foreign LCA systems revealed that although they had similar stepwise assessment methods using quantity input per process flow and use of database application, data input and result analysis were different from system to system. This is because they were developed for the general application to all industrial products, not specialized for LCA of building materials. For this reason, collected data and analysis methods can differ according to users even when the systems are used for the same purposes, which can lead to inconsistent analysis results insufficient repeatability of results and lack of objectivity in comparative analysis. Therefore, it is necessary to develop an LCA system specialized for concrete in order for concrete-related experts to easily assess environmental loading of concrete and actively apply the results to green concrete industry.

## 3. Life Cycle Assessment for Concrete

### 3.1. Overview

This study proposed a concrete LCA method including the definition of the goal and scope of concrete, inventory analysis, and impact analysis in compliance with the LCA method meeting the ISO standards. Moreover, this study broke down the steps of concrete LCA to determine input materials and energy intensities of each step for impact analysis. For the life cycle impact assessment (LCIA), this study selected six environmental impact categories, which are global warming potential (GWP), acidification potential (AP), eutrophication potential (EP), abiotic depletion potential (ADP), ozone depletion potential (ODP), and photochemical oxidant creation potential (POCP).

### 3.2. LCA Process of Concrete

#### 3.2.1. Goal and Scope Definition

**Figure 1:**
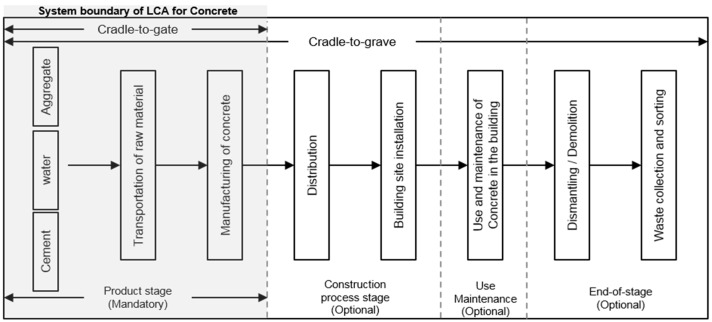
System boundary of LCA for Concrete.

The 1 m^3^ concrete was set as the functional unit on the basis of the main function to facilitate data management and application. As the system boundary for the concrete LCA (Life Cycle Assessment), the product stage of concrete was selected, as shown in Figure 1. In addition, concrete production steps were broken down into raw material extraction, transportation, and manufacturing steps, and environmental impacts of the elements involved in each step on air and water system were assessed. Because wastewater is discharged after water treatment within the factory, impact due to wastewater was not included in the assessment.

#### 3.2.2. Inventory Analysis

This study analyzed the input and output elements of the energy, raw materials, products, and waste pertaining to the scope of concrete LCA. Table 2 presents the life cycle index (LCI) database for input materials and energy sources for concrete production applied to the analysis of such materials and energy sources. The data provided by the Korea Ministry of Land, Infrastructure and Transport [15], the Ministry of Knowledge Economy, and the Ministry of Environment were used as the LCI databases for the input materials and energy sources in the present LCA. Given that LCI data vary from country to country, the database stemming from the country concerned should be used. However, because the LCI data for Ground Granulated Blast furnace Slag (GGBS), fly ash, and admixtures are not yet available in Korea, this study used foreign LCI data of Ecoinvent database [16]. LCI database of Ground Granulated Blast furnace slag (GGBS) applies the process of industrial by-product recycling. Thus, it only assesses the environmental impact of processes after the blast furnace slag is discharged as a form of a by-product. The database is derived from the amount of energy used in cooling, crushing and handling, after the slag is discharged from the blast furnace of a steel mill.

Swiss ecoinvent database is a reliable database utilized in life cycle assessments conducted in various fields in Korea. In the future, if a Korean database were developed, comparative analysis can be conducted with the ecoinvent database.

#### 3.2.3. Impact Assessment

**Figure 2:**
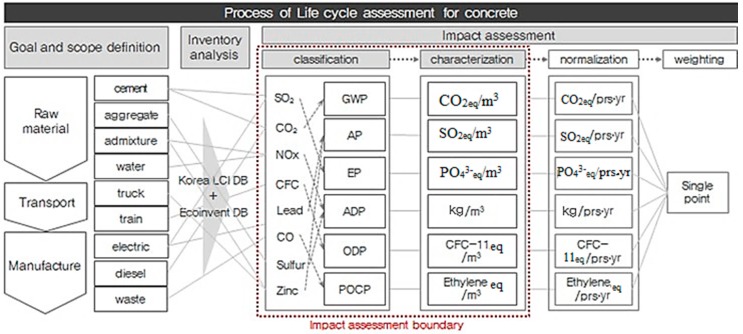
Process of Environmental impact assessment for Concrete.

Impact assessment is divided into four steps: (1) classification, in which the inventory items extracted from the inventory analysis are assigned to the corresponding impact categories; (2) characterization, in which the impact of each item classified into its impact category on each category is quantified; (3) normalization, in which the environmental impact exerted on the environmental categories are divided into local or global environmental impacts; and (4) weighting, in which relative importance among the impact categories is determined as shown in Figure 2. According to ISO 14044, the classification and characterization steps are mandatory assessment steps, and the normalization and weighting steps may be optionally assessed depending on the assessment purpose. In this study, assessment was performed for the classification and characterization steps because factors for concrete-related normalization and weighting suitable for Korean situations are yet to be developed.

The substances discharged from the raw materials and energy sources used for concrete production contaminate air and water, leading to environmental problems such as global warming, ozone depletion, photochemical oxidant creation, abiotic depletion, eutrophication, and acidification. Therefore, this study calculated concrete’s characterization value for each of these six environmental impact categories on the basis of the standard substance and impact potential unique to the category concerned. The standard substances and impact potentials for these six environmental impact categories were applied in accordance with the respective databases used in the Ministry of Environment for the eco-labeling of the Environmental Declaration of Products [17]. The classification and characterization steps of assessment were performed on the basis of the previously selected LCI database.

##### Classification

Classification is done by categorizing and compiling the inventory items according to the environmental impact categories. By linking the inventory items derived from the LCI database to the pertinent environmental impact categories and integrating them by category, the environmental impact of each inventory item can be clearly identified. For example, inventory items for GWP are GHGs such as carbon dioxide (CO_2_), methane (CH_4_), and nitrous oxide (N_2_O), with CO_2_ as the standard substance, and their respective classifications based on the Korean LCI database for OPC are 9.31 × 10^−2^ kg-CO_2_/m^3^, 1.71 × 10^−2^ kg-CH_4_/m^3^, and 1.95 × 10^−4^ kg-N_2_O/m^3^. Table 3 shows example classifications for OPC, coarse aggregate, diesel fuel, and electricity among the LCI database classification items. CO_2_, CH_4_, and N_2_O belong to the 23 GHGs specified in the Intergovernmental Panel on Climate Change (IPCC) guidelines [18], of which the standard substance is CO_2_. The classification of abiotic depletion potential (ADP) based on the standards provided by Guinee (1995) [19], takes into account a total of 89 resource items including crude oil, natural gas, and uranium (U). Acidification potential (AP) varies widely according to regional characteristics and atmospheric environments, and this study applied the AP index presented by Heijung et al. and Hauschild and Wenzel [20] applicable to all regional types. A total of 23 inventory items linked to acidification category, including sulfur dioxide (SO_2_), hydrogen sulfide (H_2_S), and hydrogen fluoride (HF), are expressed in terms of their standard substance SO_2_. Likewise, the index proposed by Heijung et al. and Hauschild and Wenzel was applied for the classification of the eutrophication potential (EP), with phosphate (PO_4_^3−^) used as the standard substance for a total of 11 inventory items including phosphate (PO_4_^3−^), ammonia (NH_3_), and nitrogen oxides (NO_x_). For the ozone depletion potential (ODP), this study applied the ODP index specified in the World Meteorological Organization (WMO) [21] for a total of 23 inventory items, including CFC-11, Halon-1301, and CFC-114, with trichloro-fluoro-methane (CFC-11) as the standard substance. For the photochemical oxidant creation potential (POCP), a total of 128 inventory items were considered, including ethylene, NMVOC, and ethanol, with ethylene being the standard substance, thereby applying the POCP index proposed by Derwent et al. [22] and Jenkin and Hayman [23].

##### Characterization

Characterization is a process of quantifying the environmental loads of inventory items itemized for each category in the classification step.

In the classification step, inventory items are assigned to their respective environmental impact categories, but there is a limitation in quantifying the potential impacts of inventory items in common metrics due to different impact potentials. Category indicator results, i.e., characterization values, are calculated in the characterization step where the environmental load (=inventory data) of each inventory item is multiplied with the characterization factor (=impact potential) unique to the impact category concerned, and the resulting environmental loads thus converted into impacts are aggregated within each impact category to yield the overall environmental impact of that category. Equation (1) expresses this process as shown in Appendix A:

(1) CI_i_ = ∑CI_i,j_ = ∑(Input material_j_ · eqv_i,j_)

where CI_i_ is the category indicator of the impact category (i), i.e., the total impact of all its inventory items assigned to the impact category (i), CI_i,j_ is the impact exerted by the inventory item (j) on the impact category (i), Input material_j_ is the impact of the j-th inventory item on the impact category (i), and eqv_i,j_ is the characterization factor of the impact category (i) [24]. Here, impact category (i) is the Global Warming Potential (GWP), Ozone Depletion Potential (ODP), Acidification Potential (AP), Abiotic Depletion Potential (ADP), Photochemical Oxidant Creation Potential (POCP) and Eutrophication Potential (EP). Taking the global warming category of OPC as an example, which involves three inventory items, CO_2_ (standard substance), CH_4_, and N_2_O, and the GWPs of CH_4_ and N_2_O are 21 kg-CO_2_/kg-CH_4_ and 310 kg-CO_2_/kg-N_2_O, respectively, as calculated by multiplying their environmental loads (index data) with the characterization factor of the global warming category of OPC. The total environmental impacts (=category indicator) on the global warming of OPC can be then obtained by adding the GWPs of the three inventory items involved. Table 4 shows the results of the category indicators of the input materials and energy sources during concrete production as a calculation example performed in this study.

(1) Global Warming Potential (GWP)

Global warming is a phenomenon that refers to the rising average surface temperature of the Earth, primarily due to the increasing level of GHG emissions. The standard substance for GWP is CO_2_. Global warming causes changes in the terrestrial and aquatic ecosystems and in coastlines due to rising sea levels.

(2) Ozone Depletion Potential (ODP)

Ozone depletion refers to the phenomenon of decreasing ozone density through the thinning of the stratospheric ozone layer (15–30 km altitude) as a result of anthropogenic pollutants. This leads to increased UV exposure of human skin, which implies a potential rise in incidence of melanoma. The standard substance for ODP is CFCs.

(3) Acidification Potential (AP)

Acidification is an environmental problem caused by acidified rivers/streams and soil due to anthropogenic air pollutants such as SO_2_, NH_3_, and NO_x_. Acidification increases mobilization and leaching behavior of heavy metals in soil and exerts adverse impacts on aquatic and terrestrial animals and plants by disturbing the food web. The standard substance for assessing AP is SO_2_.

(4) Abiotic Depletion Potential (ADP)

Input materials (natural resources) required for concrete production are classified into renewable resources, such as groundwater and wood, and nonrenewable resources, such as minerals and fossil fuels. Abiotic depletion refers to the exhaustion of nonrenewable resources and the ensuing environmental impacts.

(5) Photochemical Oxidant Creation Potential (POCP)

Photochemical oxidant creation refers to the reaction of airborne anthropogenic pollutants with sunlight that produces chemical products such as ozone (O_3_), leading to increase in ground level ozone concentration causing smog of chemical compounds adversely affecting ecosystems and hazardous to human health and crop growth. Ethylene is used as the standard substance for POCP.

(6) Eutrophication Potential (EP)

Eutrophication is a phenomenon in which inland waters are heavily loaded with excess nutrients due to chemical fertilizers or discharged wastewater, triggering rapid algal grow and red tides. The standard substance for EP is PO_4_^3−^.

## 4. Concrete Lifecycle Assessment System (CLAS)

### 4.1. Overview

This study developed the CLAS, a concrete-specific user-friendly LCA system, on the basis of the assessment technique proposed previously and LCI database.

The scope of assessment is the concrete production process (cradle to gate) deriving the results from the classification and characterization steps, which are mandatory LCA steps. Additionally, the scope of assessment was defined as the following six environmental impact categories: global warming, acidification, eutrophication, ozone depletion, abiotic depletion, and photochemical oxidant creation [25].

### 4.2. Program Composition

#### 4.2.1. Raw Material Stage

**Figure 3:**
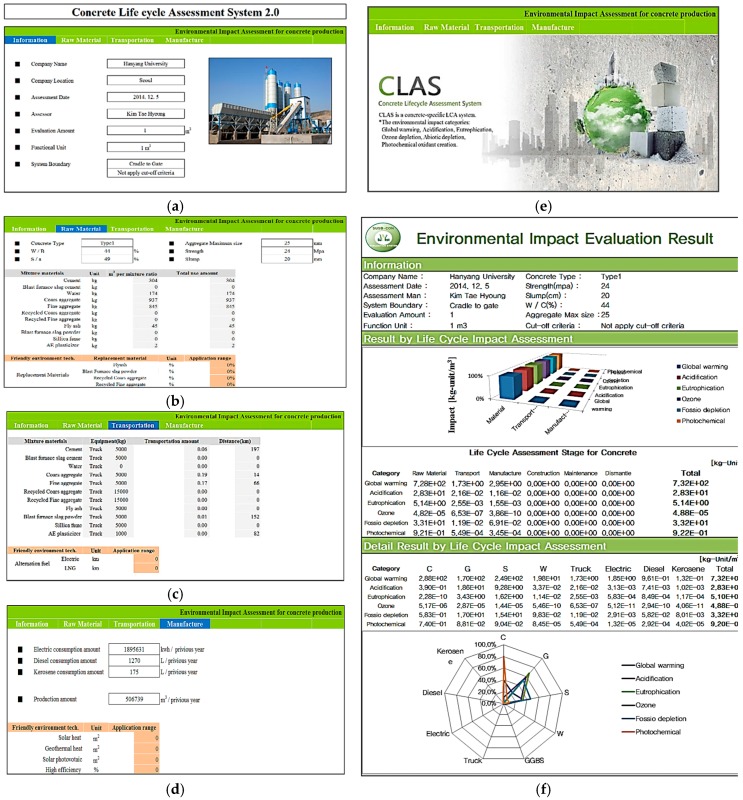
Concrete Life cycle Assessment System: (**a**) basis information sheet; (**b**) raw material stage sheet; (**c**) transportation stage sheet; (**d**) manufacture stage sheet; (**e**) main screen; and (**f**) result screen.

In this stage, the environmental impact of the raw materials used for concrete production is assessed. As shown in Figure 3b, upon entering the data for the water-binder (W/B) ratio, fine aggregate ratio (S/a), and mix amounts (kg/m^3^) of OPC, aggregates, mixing water, and admixtures of the assessment quantity of 1 m^3^ concrete, CLAS links them to the category indicators of individual raw materials embedded in the system as database and assesses the environmental impacts of concrete in six environmental impact categories. Additionally, the replacement ratio (%) of recycled aggregate can be inputted so that the environmental impacts dependent upon the replacement level can be compared.

#### 4.2.2. Transport Stage

In this stage, the environmental impacts exerted by the transport of the raw materials to the batch plant are assessed. As shown in Figure 3c, the means of transport and the travel distance inputted to the program are linked to the category indicators for transport-related variables and the relevant environmental impacts are assessed.

#### 4.2.3. Manufacture Stage

In this stage, the environmental impacts related to the energy consumption and waste generation of concrete production facilities are assessed. As shown in Figure 3d, if the values of annual concrete production of the given concrete manufacturer are entered along with the energy consumption in the form of fuel oil, water, and electricity, the energy consumption amounts of the inputted inventory items are linked to the category indicators of the individual energy sources and waste materials (solid waste/liquid waste) embedded in the system and assessed.

#### 4.2.4. Assessment Result

As shown in Figure 3f, the LCA results are outputted according to the assessment stages and input materials, revealing the assessment result for the environmental impact of each inventory item on the six environmental impact categories investigated (global warming, acidification, eutrophication, abiotic depletion, ozone depletion, and photochemical oxidant creation), broken down into concrete production steps (raw material, transport, and manufacture) and emerge source. The result outputs are displayed in radar charts that provide an intuitive graphical visualization of the magnitudes of impact of inventory items on each environmental impact category. The assessment results derived for the six environmental impact categories can also serve as database for Korean Environmental Product Declaration.

## 5. Case Study

### 5.1. Method

The newly developed Concrete Lifecycle Assessment System was applied to a cases analysis of 1 m^3^ concrete of 24 MPa strength level stemming from a batch plant located in Gyeonggi-do, South Korea. The system boundary was selected to be the production step (cradle to gate). In the case analysis, the impacts of the input materials and energy sources used for concrete production on six environmental impact categories were analyzed [26,27].

Table 5 outlines the assessment results of the raw material input amounts (kg/m^3^) required for the production of 1 m^3^ concrete (OPC: 297 kg/m^3^, coarse aggregates: 931 kg/m^3^, fine aggregates: 896 kg/m^3^, GGBS: 33 kg/m^3^, mixing water: 160 kg/m^3^, and admixtures: 2.6 kg/m^3^).

The vehicle type and travel distance for the transport of each raw material to the batch plant were identified. All the raw materials were transported by truck. Mixing water was excluded from the analysis of the transport step because on-site tap water and sewer were used.

Then, this study investigated the annual energy consumption of electricity and fuel (diesel/kerosene) required for concrete production and the quantity of produced concrete. As presented in Table 6, 3.74 kwh/m^3^ electricity, 0.2 L/m^3^ diesel fuel, and 0.03 L/m^3^ kerosene were consumed, and 3.1 kg/m^3^ waste materials were generated. Wastewater was excluded from analysis because this batch plant did not use recycling water for concrete production and discharged wastewater after on-site effluent treatment.

This study also compared the environmental impacts of different concrete mix designs in which the OPC and natural aggregates were replaced by GGBS and recycled aggregates, as shown in Table 7. Four different mix ratios (0%, 10%, 20%, and 30%) of GGBS were compared, and the same mix ratios were applied to the recycled aggregates as well in compliance with the ordinance for the mandatory use of recycled aggregate in South Korea.

### 5.2. Results

Table 8 presents the analysis results. The contributions of concrete with the strength level of 24 MPa to the environmental impact categories of global warming, acidification, eutrophication, ozone depletion, abiotic depletion, and photochemical oxidant creation potentials (GWP, AP, EP, ODP, ADP, and POCP, respectively) were assessed to amount to 309 kg-CO_2_eq/m^3^, 28.7 kg-SO_2_eq/m^3^, 5.21 kg-PO_4_^3−^eq/m^3^, 0.000049 kg-CFC-11eq/m^3^, 34 kg/m^3^, and 21 kg-ethylene-eq/m^3^, respectively. In all environmental impact categories, raw materials accounted for over 90%, with the proportions of transport and production considered insignificant [28].

**Figure 4:**
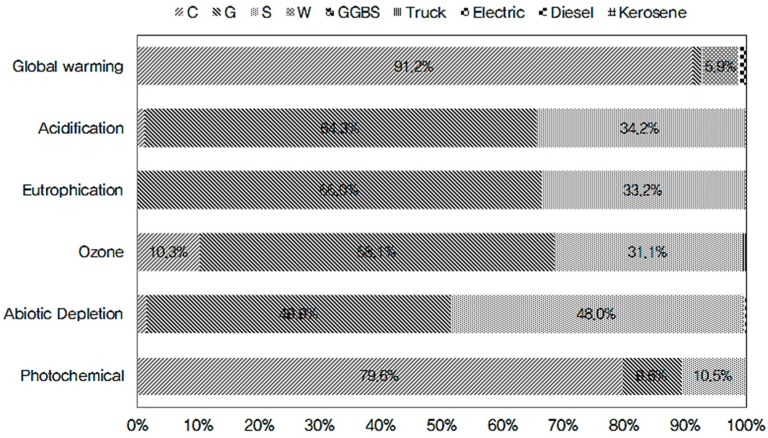
Environmental Impact Assessment Result by concrete LCA.

Figure 4 demonstrates that OPC exerted the greatest impacts on GWP and POCP, accounting for about 90% and 80%, respectively, of the entire environmental impact categories. This is assumed to be ascribable to the emissions of sulfur dioxide and sulfuric acid due to the use of dynamites comprising sulfuric acid, nitric acid, and sulfur when mining limestone and iron ore, which are primary components of OPC. Furthermore, NO_x_ and PO_4_^3−^ are emitted through the power consumption when crushing the extracted ore and clinker. Clinkering is the work unit that emits the largest amount of energy input and release of substances with environmental impact. When producing clinker in a rotary kiln, the temperature inside the kiln is raised to 1000–1450 °C, using various fuel sources such as Bunker C oil, coal, waste tires, and waste plastics, from which substances such as CO_2_ and NH_3_ are emitted. Most of these fuels are based on crude oil, which contains the elements carbon and hydrogen as primary components and additionally nitrogen, oxygen, and sulfur compounds. Sulfur and nitrogen compounds also cause corrosion and stench.

Of the raw materials, coarse aggregates show the highest contribution to AP, EP, ODP, and ADP, accounting for about 65%, 65%, 60%, and 50%, respectively. This is primarily attributable to the lubricants and dynamites used for logging and rock blasting because their primary components are coal minerals and sulfuric acid, respectively, which emit substances with high contributions to AP, EP, and ODP, such as SO_2_, H_2_SO_4_, and NO_3_. Crushing blasted rocks also involves diesel fuel and electric power, emitting NH_3_, NH_4_+, PO_4_^3−^, NO_x_, etc. These impact substances emitted by diesel fuel and electric power have similar tendencies, as similar substances are emitted from crude oil, which is the main component of diesel fuel, and coal used as fuel for thermoelectric power generation.

#### 5.2.1. Analysis of Environmental Impacts According to the Mix Ratio of Admixtures

**Figure 5:**
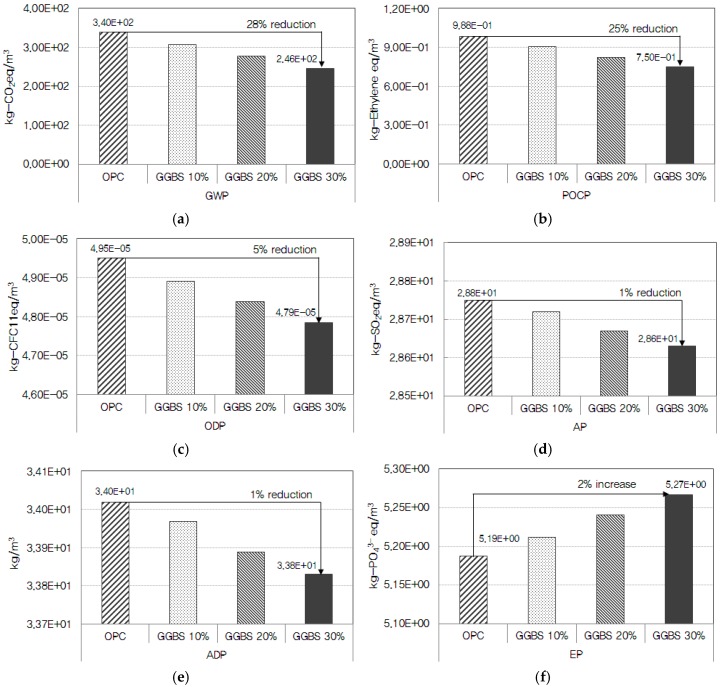
Analysis of environmental impacts according to the mix ratio of GGBS: (**a**) global warming potential (GWP); (**b**) photochemical ozone creation potential (POCP); (**c**) ozone depletion potential (ODP); (**d**) acidification potential (AP); (**e**) abiotic depletion potential (ADP); and (**f**) eutrophication potential (EP).

Figure 5 depicts the analysis results of environmental impacts according to the mix ratio of ground granulated blast-furnace slag (GGBS) in the concrete of 24 MPa strength level. GGBS is produced by crushing and mixing blast furnace slag (by-product of iron ore) and natural gypsum, followed by cooling. All GGBS production facilities were found to be fueled by electricity and diesel, which emitted as many as 45 substances including CO_2_, CH_4_, S, NH_3_, and NO_x_. The higher the mix ratio of GGBS was, the lower the contribution to GWP, AP, ODP, ADP, and POCP became, while the impact on EP increased. The impacts on GWP and POCP were assessed to be reduced by 10%–28% as compared to OPC, as the GGBS mix ratio increased from 10% to 20% and 30%. This was analyzed to be ascribable to the reduced emissions of CO_2_, CH_4_, N_2_O, CO, and S that contribute to GWP and POCP. As the mix ratio of GGBS increased, impacts on AP, ODP, and ADP were found to be reduced by about 1%–5% as compared to OPC. This was due to the reduction of NO_x_, SO_2_, halon, CFC, soft coal, hard coal, and crude oil in the GGBS production process compared to OPC, albeit to a negligible degree. Of the six environmental impact categories, the impact on EP was analyzed to increase by about 1%–2% compared to OPC, as the mix ratio of GGBS. This is due to the increased emissions of NH_4_, NH_3_, NO_3_, N_2_, PO_4_^3−^, which greatly contribute to EP, in the GGBS production process compared to OPC production [29,30].

#### 5.2.2. Analysis of Environmental Impacts According to the Mix Ratio of Recycled Aggregate

**Figure 6:**
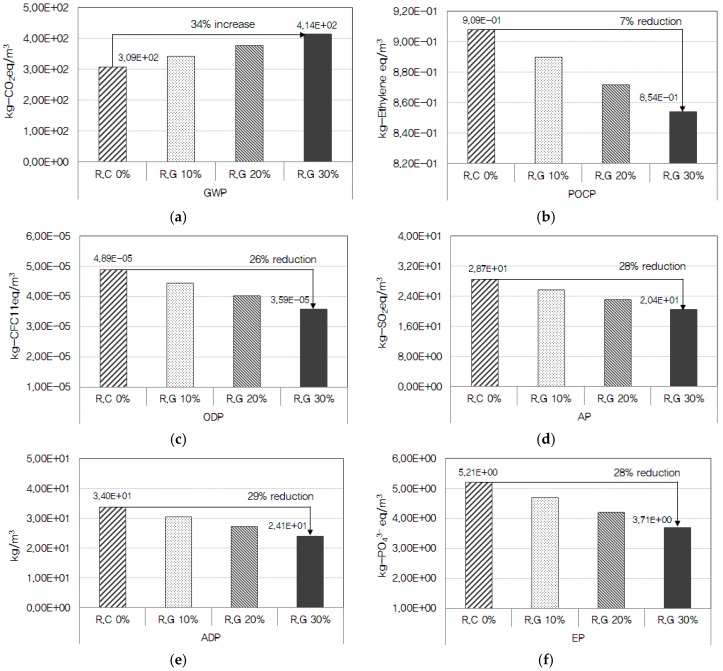
Analysis of environmental impacts according to the mix ratio of recycled aggregate: (**a**) global warming potential (GWP); (**b**) photochemical ozone creation potential (POCP); (**c**) ozone depletion potential (ODP); (**d**) acidification potential (AP); (**e**) abiotic depletion potential (ADP); and (**f**) eutrophication potential (EP).

As shown in Figure 6, this study analyzed the environmental impacts according to the mix ratio of recycled aggregate in the concrete of *2*4 MPa strength level. Recycled aggregates are produced with construction wastes from demolition or dismantling of buildings and other structures (roads, bridges, etc.) through physical or chemical processes of shredding, separating, sorting, and grain size optimization. All equipment used for the production of recycled aggregate was found to be fueled by electricity and diesel, which emitted 45 substances including CO_2_, CH_4_, S, NH_3_, and NO_x_. The higher the mix ratio of recycled aggregate was, the lower the contribution to AP, EP, ODP, ADP, and POCP became, while the impact on GWP increased. The impacts on AP, EP, ODP, and ADP were assessed to be reduced by about 9%–29% as compared to concrete using only natural aggregates, as the recycled aggregate mix ratio increased from 10% to 20% and 30%. This was analyzed to be ascribable to the reduced emissions of NO_x_, NH_3_, SO_2_, NH_4_, halon, and CFC (from soft coal, hard coal, and crude oil), which contribute to AP, EP, ODP, and ADP, in the production process of recycled aggregates compared to natural aggregates. As the mix ratio of recycled aggregate increased, impact on POCP was found to decrease by about 2%–7% compared to concrete using only natural aggregates. This was due to the reduction of CH_4_, CO, S, and C_4_H_10_ emission in the recycled aggregate production process compared to natural aggregates, but the differences were not significant [31,32].

Of the six environmental impact categories, the impact on GWP was analyzed to increase up to 11%–34% compared to concrete with only natural aggregates, as the mix ratio of recycled aggregates increased. This is due to the increased emissions of CO_2_, CH_4_, and N_2_O, which primarily contribute to GWP, in the recycled aggregate production process compared to natural aggregates proposed.

## 6. Discussion

This study evaluates the environmental impact of mixing recycled aggregate and ground granulated blast furnace slag on concrete.

Currently, GWP, AP, EP, ODP, ADP, and POCP values, derived using mid-point method applied throughout the life cycle environmental impact assessment, are not comparable to GWP and ADP values. Moreover, it cannot be claimed that ODP has less impact on the ecological environment because its values are lower than those of GWP and ADP.

This is because each environmental impact category has a different unit of measurement and does not allow for an integrated and comparative analysis.

However, selection and quantitative analysis of high environmentally-performing concrete is possible using end-point methodology, which is one of the life cycle impact assessment techniques. End-point methodology utilizes research results of various natural sciences, such as toxicology and human epidemiology, to compute the degree of damage an environmental impact substance has on human health and ecology. This is used to determine its weighted value for conversion and representation as an index or an environmental cost value.

As a result of analysis using end-point methodology, acidification (AP) had the largest portion of the combined environmental costs. This indicates that the degree of damage acidification has on human health and ecosystem is the most significant. In addition, environmental cost of abiotic depletion (ADP) and photochemical oxidant creation (POCP) were also significant. In contrast, environmental cost of global warming (GWP) was less than those of AP, ADP and POCP. Thus, using end-point methodology will be of great importance in subsequent environmental performance evaluations of concrete.

## 7. Conclusions

In this paper, this study proposed a concrete-specific LCA technique tailored to Korean situations by adapting the ISO standards that can assess environmental impacts on the bases of the input data on concrete mix design, means of transport, travel distance, energy consumption in the batch plant, etc.

This study established a database of category indicators (characterization values) of the raw materials for concrete mix and energy sources required for the concrete production process on the basis of the standard substance and impact potential (characterization factor) unique to each of the six environmental impact categories (global warming, acidification, eutrophication, ozone depletion, abiotic depletion, and photochemical oxidant creation).

The case analysis of 24-MPa concrete performed using the proposed CLAS yielded the assessment results of 309 kg-CO_2_ eq/m^3^, 28.7 kg-SO_2_ eq/m^3^, 5.21 kg-PO_4_^3−^ eq/m^3^, 0.000049 kg-CFC-11 eq/m^3^, 34 kg/m^3^, and 21 kg-Ethylene eq/m^3^ for global warming, acidification, eutrophication, ozone depletion, abiotic depletion, and photochemical oxidant creation potentials (GWP, AP, EP, ODP, ADP, and POCP), respectively.

Of the six environmental impact categories, ordinary Portland cement (OPC) exerted greatest impacts on GWP and POCP, and aggregates on AP, EP, ODP, and ADP.

While GWP decreased in proportion to the increase in GGBS mix ratio in concrete mix design, the remaining five environmental impact categories showed negligible reduction or increase. In contrast, as the mix ratio of recycled aggregate increased, GWP increased and AP, EP, ODP, ADP, and POCP decreased.

These case analysis results allow the assumption that single-category environmental impact assessment cannot yield any reliable assessment results regarding the eco-sustainability of concrete, which requires multi-category assessment. The results derived in this study are not representative of the impact potentials of concrete of all strength levels, and further analyses should be performed for different strength levels in order to establish the standard range of impact potentials pertaining to various strength levels.

## Figures and Tables

**Table 1:** Survey of Life Cycle Assessment (LCA) program.

Division		Scope of Environmental Impact Assessment							
Program	Nation	GWP	AP	EP	ADP	ODP	POCP	ET	HT
Total	Korea	■	■	■	■	■	■	-	-
Pass	Korea	■	■	■	■	■	■	-	-
Cool	Korea	■	-	-	-	-	-	-	-
Bees	U.S.	■	■	■	■	■	■	■	■
Athena	Canada	■	■	■	■	■	■	■	■
Gabi Build-it	Germany	■	-	-	-	-	-	-	-
Sima-Pro	Netherlands	■	■	■	■	■	■	■	■
This research	Korea	■	■	■	■	■	■	-	-

GWP: Global Warming Potential; AP: Acidification Potential; ADP: Abiotic Depletion Potential; EP: Eutrophication Potential; ODP: Ozone Depletion Potential; ET: Eco Toxicity; POCP: Photochemical Ozone Creation Potential; HT: Human Toxicity. ■: included, -: not included.

**Table 2:** LCI Database.

Division		Reference	Nation
Raw material	Cement	National LCI	Korea
	Coarse aggregate	National LCI	Korea
	Fine aggregate	National LCI	Korea
	Blast furnace slag	Ecoinvent	Switzerland
	Fly ash	Ecoinvent	Switzerland
	Water	National LCI	Korea
	Chemical admixture	Ecoinvent	Switzerland
Energy	Electric	National LCI	Korea
	Diesel	National LCI	Korea
	Kerosene	National LCI	Korea
Transportation	Truck	National LCI	Korea
	Train	National LCI	Korea

**Table 3:** Classification value of Composition material for concrete.

Inventory List	Environmental Impact Categories						Composition Material	
	GWP	ADP	AP	EP	ODP	POCP	Cement	Aggregate
Ammonia (NH_3_)	-	-	■	■	-	-	-	6.95 × 10^−7^
Carbon dioxide (CO_2_)	■	-	-	-	-	-	9.31 × 10^−1^	3.40 × 10^−1^
CFC-11	■	-	-	-	■	-	2.05 × 10^−9^	4.02 × 10^−13^
Ethylene	-	-	-	-	-	■	-	-
Methane (CH_4_)	■	-	-	-	-	■	1.71 × 10^−2^	5.57 × 10^−4^
Nitrogen oxides (NO_x_)	-	-	■	■	-	-	-	1.38 × 10^−6^
Sulfur dioxide (SO_2_)	-	-	■	-	-	■	1.27 × 10^−2^	4.42 × 10^−4^
Phosphate (PO_4_^3−^)	-	-	-	■	-	-	-	4.22 × 10^−8^

■: included, -: not included.

**Table 4:** Characterization value example of Composition material for concrete.

Composition Material	Unit	Environmental Impact Categories			
		GWP	AP	EP	POCP
		kg-CO_2eq_/Unit	kg-SO_2eq_/Unit	kg-PO_4_^3−^_eq_/Unit	kg-Ethylene_eq_/Unit
Cement	kg	0.948	0.00128	0.000134	0.00243
Fine aggregate	kg	0.00149	0.011	0.00192	0.000107
Fly ash	kg	0.015	0.000116	0.0000694	0.0000657
Water	kg	0.114	0.000194	0.0000657	0.000000486
Chemical admixture	kg	0.0129	0.0000248	0.000319	0.000209

**Table 5:** Concrete mix design and transportation distance.

	OPC	W	G	S	GGBS	AE
Mix design (kg/m^3^)	297	160	931	896	33	2.6
Transport distance (km)	201	-	14	66	122	90

OPC: Ordinary Portland cement W: Water GGBS: Granulated ground blast furnace slag; G: Coarse aggregate S: Fine aggregate AE: Chemical admixture.

**Table 6:** Energy consumption of manufacturing process.

Production of Concrete (m^3^/year)	Energy Consumption			Waste (ton/year)
	Electric (kwh/year)	Diesel (L/year)	Kerosene (L/year)	
506,739	1,895,631	101,348	15,202	1570

**Table 7:** Classification of concrete mix design.

Strength (MPa)	Mix Design (kg/m^3^)							
	OPC	GGBS	G	R.G	S	R.S	W	AE
24	100%	0%	Same					
	90%	10%						
	80%	20%						
	70%	30%						
	90%	10%	100%	0%	100%	0%	Same	
			90%	10%	90%	10%		
			80%	20%	80%	20%		
			70%	30%	70%	30%		

OPC: Ordinary Portland cement W: Water GBS: Granulated ground blast furnace slag G: Coarse aggregate S: Fine aggregate AE: Chemical admixture; R.G: Recycled coarse aggregate R.S: Recycled fine aggregate.

**Table 8:** Environmental impact assessment result.

Division	Production Stage (Cradle to Gate)			Total
	Raw Material	Transportation	Manufacture	
GWP (kg-CO_2eq_/m^3^)	3.05 × 10^2^	5.95 × 10^−1^	2.95	3.09 × 10^2^
AP (kg-SO_2eq_/m^3^)	2.87 × 10	7.45 × 10^−3^	1.16 × 10^−2^	2.87 × 10
EP (kg-PO_4_^3−^_eq_/m^3^)	5.21	8.76 × 10^−4^	1.55 × 10^−3^	5.21
ODP (kg-CFC-11_eq_/m^3^)	4.87 × 10^−5^	2.25 × 10^−7^	3.86 × 10^−10^	4.90 × 10^−5^
ADP (kg/m^3^)	3.39 × 10	4.08 × 10^−3^	6.91 × 10^−2^	3.40
POCP (kg-Ethylene_eq_/m^3^)	9.08 × 10^−1^	1.89 × 10^−4^	3.45 × 10^−4^	9.08 × 10^−1^

## Appendix A

(1) Example of GWP assessment for concrete:
  Cement GWP = Input material(i) × GWP(i) (Table 4)
    = (240 kg/m^3^ × 0.9 CO_2_ factor (kg-CO_2_/kg) = 216 kg-CO_2_ eq/kg
(2) Example of ODP assessment for concrete:
  Cement ODP = Input material(i) × ODP(i) (Table 4)
    = (240 kg/m^3^ × 0.000000017 CFCs factor (kg-CFCs/kg) = 0.00000408 kg-CFCs eq/kg
(3) Example of AP assessment for concrete:
  Cement AP = Input material(i) × AP(i) (Table 4)
    = (240 kg/m^3^ × 0.0128 SO_2_ factor (kg-SO_2_/kg) = 3.072 kg-SO_2_ eq/kg
(4) Example of POCP assessment for concrete:
  Cement POCP = Input material(i) × POCP(i) (Table 4)
    = (240 kg/m^3^ × 0.0243 Ethylene factor (kg-Ethylene/kg) = 5.832 kg-Ethylene eq/kg
(5) Example of ADP assessment for concrete:
  Cement ADP = Input material(i) × ADP(i) (Table 4)
    = (240 kg/m^3^ × 0.0192 Antimony factor (kg-Antimony/kg) = 4.608 kg-Antimony eq/kg
(6) Example of EP assessment for concrete:
  Cement EP = Input material(i) × EP(i) (Table 4)
    = (240 kg/m^3^ × 0.000134 PO_4_^3−^ factor (kg-PO_4_^3−^/kg) = 0.03216 kg-PO_4_^3−^eq/kg

## References

[1] Building Research Establishment (2011). Methodology for Environmental Profiles of Construction Products-Product Category Rules for Type 3 Environmental Product Declaration of Construction Product.

[2] (2013). International EPD System, Product Category Rules-Concrete. http://environdec.com/.

[3] (2014). ISO 13315–2 (Environmental Management for Concrete and Concrete Structures)—Part 2: System Boundary and Inventory Data. https://www.iso.org/obp/ui/#iso:std:iso:13315:-2:ed-1:v1:en.

[4] International Organization for Standardization (2006). ISO 14025, Environmental Labels and Declarations-Principles and Procedures.

[5] International Organization for Standardization (2006). ISO 14044, Life Cycle Assessment-Requirements and Guidelines.

[6] International Organization for Standardization (2007). ISO 21930, Sustainability in Building Construction-Environmental Declaration of Building Products.

[7] Environmental Declaration Office Life Cycle Impact Assessment. http://www.edp.or.kr/lci/total.asp.

[8] Korea National Cleaner Production Center. http://www.kncpc.or.kr/main/main.asp.

[9] COOL Program of Carbon Footprint Labeling. http://www.epd.or.kr/information/data.asp?bbs_code=6&mode=view&bbs_idx=2172&page=1&search_type=0&search_word=&bbs_class=.

[10] Korea Environmental Industry and Technology Institute, National Life Cycle Index Database Information Network. http://www.edp.or.kr.

[11] Athena Sustainable Materials Institute The Athena Sustainable Materials Institute Is a Non-Profit Research Collaborative Bringing Life Cycle Assessment to the Construction Sector. http://www.athenasmi.org.

[12] Thinkstep Gabi. http://www.gabi-software.com.

[13] National Institute of Standards and Technology BEES. http://www.nist.gov/el/economics/BEESSoftware.cfm.

[14] Simapro. http://www.simapro.co.uk/.

[15] Ministry of Land, Transport and Maritime Affairs of the Korean Government (2008). National D/B for Environmental Information of Building Products.

[16] Ecoinvent. http://www.ecoinvent.org/database.

[17] Environmental Declaration Office. http://www.edp.or.kr/edp/edp_intro.asp.

[18] (2006). IPCC Guidelines for National Greenhouse Gas Inventories.

[19] Guinee J.B. (1995). Development of a Methodology for the Environmental Life Cycle Assessment of Products: With a Case Study on Margarines. Ph.D. Thesis.

[20] Heijungs R., Guinée J.B., Huppes G., Lamkreijer R.M., Udo de Haes H.A., Wegener Sleeswijk A., Ansems A.M.M., Eggels P.G., van Duin R., de Goede H.P. (1992). Environmental Life Cycle Assessment of Products: Guide (Part 1) and Background (Part 2).

[21] World Metrological Organization (WMO) (1991). Scientific Assessment of Ozone Depletion: Global Ozone Research and Monitoring Project.

[22] Derwent R., Jenkin M.S., Piling M.J. (1998). Photochemical ozone creation potentials for organic compounds in Northwest Europe calculated with a master chemical mechanism. Atmos. Environ..

[23] Jenkin M., Hayman G. (1999). Photochemical Ozone Creation Potentials for oxygenated volatile organic compounds: Sensitivity to variation is in kinetic and mechanistic parameters. Atmos. Environ..

[24] Youngche H., Sangwon S., Sangwon H., Keonmo L. (2000). Determination of normalization values for Korean eco-indicator. Korean Soc. Life Cycle Assess..

[25] Taehyoung K., Sungho T., Seongjun R. (2013). Assessment of the CO_2_ emission and cost reduction performance of a low-carbon-emission concrete mix design using an optimal mix design system. Renew. Sustain. Energy Rev..

[26] Sungho T., Sungwoo S., Jeehwan W., Seongjun R. (2011). The Development of Environmental Load Evaluation System of a Standard Korean Apartment House. Renew. Sustain. Energy Rev..

[27] Lee K.H., Tae S.H., Shin S.W. (2009). Development of a life cycle assessment program for building (SUSB-LCA) in South Korea. Renew. Sustain. Energy Rev..

[28] Tae S.H., Shin S.W., Lee K.H. (2008). The Environmental Load Reduction Effect of the Reinforced Concrete Structure Using High-Strength Concrete. Korea Inst. Ecol. Archit. Environ..

[29] Park J.H., Tae S.H., Kim T.H. (2012). Life cycle CO_2_ assessment of concrete by compressive strength on construction site in Korea. Renew. Sustain. Energy Rev..

[30] Kim T.H., Tae S.H., Roh S.J., Kim R.H. (2013). Life Cycle Assessment for Carbon Emission Impact Analysis of Concrete Mixing Ground Granulated Blast-furnace Slag (GGBS). Archit. Inst. Korea.

[31] Jung J.S., Lee J.S., An Y.J., Lee K.H., Bae K.S. (2008). An Analysis of Emission of Carbon Dioxide from Recycling of Waste Concrete. Archit. Inst. Korea.

[32] Jung C.J., Chang B.K., Lee J.H. (1996). Experimental Study on the Recycling of Concrete Sludge Produced in Ready-Mixed-Concrete Works. Korea Soc. Waste Manag..

